# Androgens’ effects on cerebrovascular function in health and disease

**DOI:** 10.1186/s13293-020-00309-4

**Published:** 2020-06-30

**Authors:** Charly Abi-Ghanem, Lisa S. Robison, Kristen L. Zuloaga

**Affiliations:** Department of Neuroscience & Experimental Therapeutics, Albany Medical College, 47 New Scotland Avenue, MC-136, Albany, NY 12208 USA

**Keywords:** Androgens, Cerebral vasculature, Stroke, Vascular contributions to cognitive impairment and dementia, Cerebrovascular disease, Testosterone, Dihydrotestosterone, Hormone therapy, Endothelial, Blood-brain barrier

## Abstract

Androgens affect the cerebral vasculature and may contribute to sex differences in cerebrovascular diseases. Men are at a greater risk for stroke and vascular contributions to cognitive impairment and dementia (VCID) compared to women throughout much of the lifespan. The cerebral vasculature is a target for direct androgen actions, as it expresses several sex steroid receptors and metabolizing enzymes. Androgens’ actions on the cerebral vasculature are complex, as they have been shown to have both protective and detrimental effects, depending on factors such as age, dose, and disease state. When administered chronically, androgens are shown to be pro-angiogenic, promote vasoconstriction, and influence blood-brain barrier permeability. In addition to these direct effects of androgens on the cerebral vasculature, androgens also influence other vascular risk factors that may contribute to sex differences in cerebrovascular diseases. In men, low androgen levels have been linked to metabolic and cardiovascular diseases including hypertension, diabetes, hyperlipidemia, and obesity, which greatly increase the risk of stroke and VCID. Thus, a better understanding of androgens’ interactions with the cerebral vasculature under physiological and pathological conditions is of key importance.

## Background

Despite the fact that the brain only makes up 2% of the body’s mass, it utilizes 20% of the oxygen and nutrients. These great metabolic needs are fulfilled by its unique vasculature that, in combination with neural cells, forms a highly specialized neurovascular unit (NVU). As any other vascular system, the NVU is subject to modulation by androgens through several direct and indirect mechanisms. The purpose of this review is to highlight the effects of androgens on the cerebral vasculature under physiological and pathological conditions, such as cerebrovascular diseases (stroke and vascular contributions to cognitive impairment and dementia), which are both highly prevalent and life-threatening. Very little is known about the effects of androgens in the female cerebral vasculature. Thus, all studies presented here are in males unless otherwise noted. The effects of androgens on cerebral vessels in females are a major gap in knowledge in the field.

### The cerebral vasculature

A continuous and well-regulated blood supply is essential for normal brain function. Indeed, the brain lacks energy reserves but has high energy requirements. Therefore, partial or complete interruption of cerebral blood flow (CBF) can have deleterious consequences ranging from subclinical microinfarcts, to stroke, cognitive impairment, dementia, or even death.

The brain vascularization pattern differs from that of other major organs, such as the liver and kidneys, as it is vascularized from the outside-in [1]. Cerebral vessels arise from the circle of Willis (formed by the confluence of the internal carotid arteries and the basilar artery), envelop it (pial arteries), and then dive into the brain matter (penetrating arterioles), where they form a rich anastomotic network of capillaries. Each blood vessel is formed by a monolayer of endothelial cells (ECs) surrounded by layers of vascular smooth muscle cells (VSM). VSM contractions alter vessel diameter and therefore regulate blood flow. The larger the vessel, the greater the number of VSM layers, ranging from two to three for arterioles up to 20 layers for large arteries. Pericytes replace VSMs on capillaries [2, 3]. Pial arteries and smaller arterioles branching from them are surrounded by the perivascular space (Virchow-Robin space), an extension of the subarachnoid space filled with cerebrospinal fluid. The perivascular space is bound by the vascular basement membrane and the basement membrane of the glia limitans and is important for the removal of waste products, such as amyloid beta [4–7]. Deeper arterioles become smaller in diameter (< 100 μm) and are devoid of the perivascular space. Their basement membrane is in direct contact with that of the glial cells and is enveloped by the astrocytic end-feet. For a thorough overview of the cerebral vasculature, we refer readers to Cipolla 2009 [8]. In the following sections, we will explore how androgens can influence this extensive cerebrovascular network.

## Androgens

### Synthesis and metabolism

Androgens are natural or synthetic steroids that are agonists of the androgen receptor (AR). They are synthesized through a serial metabolism of cholesterol by several enzymes present in the mitochondrial membrane and the endoplasmic reticulum.

Most circulating androgens (predominantly testosterone (T) and 5α-dihydrotestosterone (DHT)) are synthesized in gonads and adrenal glands. Androgens readily cross the blood-brain barrier (BBB) but can be locally synthesized or metabolized in several organs such as muscle, the central nervous system (CNS), and the cerebrovasculature. Indeed, T can be metabolized by 5α–reductase into DHT, a more potent androgen receptor agonist. Moreover, both T and DHT can be further metabolized into hormones that activate the estrogen receptor (ER). Aromatase converts T into 17β-estradiol, and DHT can be metabolized into 5α-androstane-3β, 17β-diol (3β-diol), which activates estrogen receptor β (ERβ) [9].

### Androgens’ modes of action

Androgens’ classical signaling pathway occurs through the AR, a nuclear receptor that acts as a ligand-activated transcription factor. At least 7 splice variants of AR have been identified [10]. AR is normally sequestered in the cell cytoplasm by a complex of proteins including heat shock proteins (e.g., HSP90). Upon binding to its ligand, AR is liberated, homodimerizes, is phosphorylated, and translocates into the nucleus where it binds its target DNA on the level of androgen response elements. It then recruits a series of transcription activators or repressors, influencing the expression of target genes. This genomic effect is a relatively slow process that requires several hours. It is noteworthy that AR potency is inversely linked to a polymorphism of trinucleotide CAG expansion in the first exon, with longer repeats leading to a decrease of the receptor’s transactivation function [11]. This is an important factor to consider during T administration, as individuals with shorter AR gene CAG expansions would yield a greater response than those with a longer repeat [12].

Non-genomic, fast-acting, actions of androgens have also been described. These are mediated by a membrane-bound AR, in addition to other receptors such as G-protein-coupled receptor C6AG (PRC6A) and Zrt- and Irt-like protein 9 (ZIP9) [13]. Some of the non-genomic actions of androgens on the vasculature include increases in intracellular calcium and protein kinase C activation; increases in cAMP, activation of phosphatidylinositol-3 kinase/Src pathway; and increases in nitric oxide (NO) production [13].

Of note, both T and DHT can be metabolized into ER agonists, as previously mentioned. Two isoforms of nuclear ERs have been identified, ERα and ERβ, and at least five splice variants have been identified for ERβ [14]. Estrogens can also act through non-genomic actions mediated by membrane-bound ERα (mERα) and ERβ (mERβ), as well as the membrane-bound G-protein-coupled estrogen receptor GPER1 (also known as GPR30) [15–17]. Even though estrogens’ effects on the NVU are the most well described [18], we will focus on androgens in this review and would like to refer the readers to Robison et al. 2019 for more details on estrogen effects [19].

Finally, T route of administration has been shown to have an impact on its effects [20, 21]. When compared to intramuscular and transdermal administration, oral T treatment results in higher cardiovascular risk. Further, serum DHT levels (but not T) were greater when T administration was transdermal versus intramuscular, possibly due to metabolism by 5α-reductase in the skin [20].

### Cerebrovascular expression of receptors and metabolizing enzymes

**Fig. 1:**
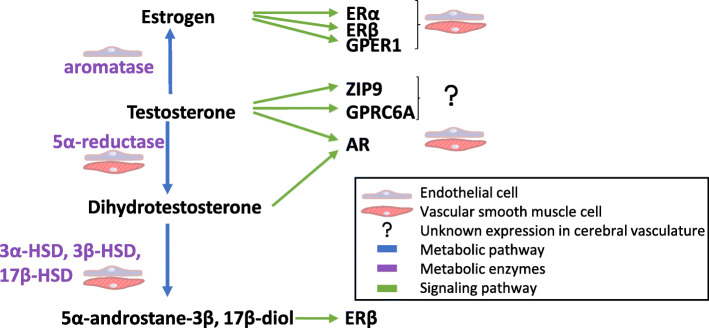
Androgen metabolism and signaling in the cerebral vasculature. Signaling pathways are indicated using green arrows. Metabolic pathways are indicated using blue arrows, and metabolizing enzymes are in purple. Confirmed expression in cells of the cerebral vasculature is indicated by cartoon cell types (see key). AR, androgen receptor; ER, estrogen receptor; HSD, Hydroxysteroid dehydrogenase; ZIP9, Zrt- and Irt-like protein 9; GPRC6a, G-protein-coupled receptor C6A; GPER1, G-protein-coupled estrogen receptor

**Fig. 2:**
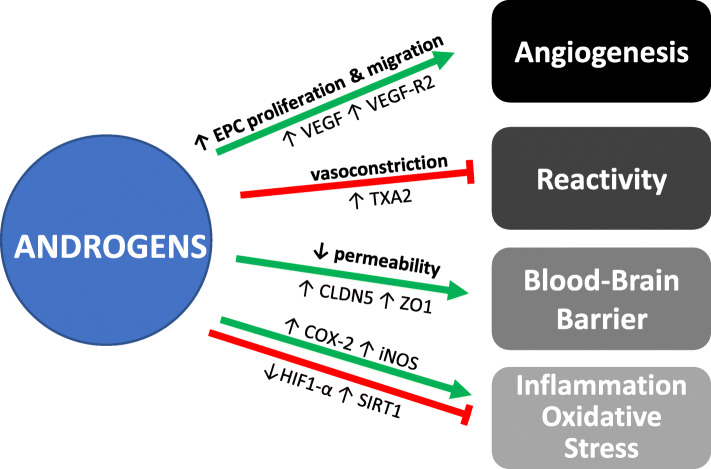
Effects of androgens on cerebrovascular function. Green arrows represent promotion of the function, while red lines with rectangles at ends represent inhibition of the function. Mechanisms by which these effects have been shown to occur in the cerebral vasculature are listed along the lines. COX-2, cyclooxygenase 2; CLDN5, claudin-5; EPC, endothelial progenitor cell; HIF-1α, hypoxia- inducible factor 1-alpha; iNOS, inducible nitric oxide synthase; TXA2, thromboxane A2; SRT1, Sirtuin 1; VCID, vascular contributions to cognitive impairment and dementia; VEGF, vascular endothelial growth factor; VEGF-R2, vascular endothelial growth factor receptor 2; ZO1, tight junction protein ZO-1

The cerebral vasculature of both rodents and humans express sex steroid receptors and metabolizing enzymes (Fig. 1). Indeed, male human brain microvascular ECs [22] and human brain VSM cells [23] express ERα, ERβ, and AR, and many sex hormone metabolizing enzymes including 3β-hydroxysteroid dehydrogenase (HSD), 3α-HSD, 17β-HSD, and CYP7B1. In the rodent cerebral vasculature, ERα, AR, GPER1, and 5α-reductase are expressed in both VSMs and ECs [24–27], while aromatase and ERβ have only been reported in ECs in the rodent cerebral vasculature [27, 28]. Data on aromatase expression in human brain endothelial cells is lacking, as well as data on the expression of the majority of these receptors and enzymes in pericytes. The expression of steroid receptors and metabolizing enzymes in the cerebral vasculature (Fig. 1) make it a potential target for sex hormone actions (Table 1, Fig. 2).

**Table 1:** Effects of androgens on functions of the cerebral vasculature

Function	Subjects/cells	Androgen	Results	Reference
Angiogenesis	EPC (healthy male donors)	Methyltrienolone (synthetic androgen)	Dose-dependent increase in proliferation, migration and colony formation. Effect is AR-dependent (blocked by flutamide).	Foresta et al. [29]
Angiogenesis	EPC (healthy male donors)	DHT	Dose- and time-dependent increase in VEGF and proliferative, migratory, and adhesive abilities of EPCs. Via RhoA/ROCK pathway.	Zhang et al. [30]
Angiogenesis	EPC (healthy male donors)	DHT	Dose- and time-dependent increase in proliferative activity and adhesive ability of EPCs. The PI3-K/Akt pathway plays a role.	Liu et al. [31]
Angiogenesis	Healthy adult males	N/A	T/E2 ratio negatively correlated with number of EPCs.	Fadini et al. [32]
Angiogenesis	EPC (healthy male donors)	T or DHT	No effect on expansion and function of late EPCs; increase generation of early EPCs	Fadini et al. [32]
Angiogenesis	Adult male SD rats (intact & GDX)	T or DHT replacement	T & DHT replacement unable to restore reduced EPC levels in GDX males	Fadini et al. [32]
Angiogenesis	Adult female canaries	T	Increased angiogenesis and endothelial BDNF, VEGF, and VEGF-R in the brain	Louissaint et al. [33]
Cerebrovascular reactivity	Adult male Fisher 344 rats (intact & GDX)	T replacement	Endothelium-dependent myogenic tone is reduced in GDX rats, which is NOS-independent and reversed by T replacement.	Geary et al. [34]
Cerebrovascular reactivity	Adult male Fisher 344 rats (GDX)	T replacement	T replacement increased vascular tone in endothelium-dependent manner of isolated MCA, independent of COX, NOS, and endothelin. Effect may be through suppression of EDHF-like vasodilator.	Gonzales et al. [35]
Cerebrovascular reactivity	Adult male Fisher 344 rats (GDX)	T replacement	T replacement increased vascular tone of MCA, likely by increasing synthesis of thromboxane A2.	Gonzales et al. 2005 [36]
BBB function and inflammation	Adult male C57BL/6J mice (intact & GDX)	T replacement	GDX increased BBB permeability and inflammation; reduced EC TJ proteins. Effects reversed by T replacement.	Atallah et al. [37]
Blood-spinal cord barrier	Young adult male Wistar rats	T	T treatment decreased some TJ proteins in spinal cord and increased P-gp expression.	Nierwinska et al. [38]
Inflammation	Adult male Wistar rats (GDX)	DHT replacement	DHT stimulated CBV inflammation by increasing COX-2, iNOS, and NFkB levels in cerebral arteries (ex vivo and in vivo). Effect is AR-dependent (blocked by flutamide).	Gonzales et al. [39]
Inflammation	Adult male Wistar rats (GDX)	DHT (in vivo or ex vivo)	DHT increases COX-2 under normoxia but decreases COX-2 under hypoxia in cerebral arteries. DHT blunts HIF-1a following hypoxia.	Zuloaga & Gonzales [40]
Inflammation	Primary human brain VSMC	DHT	DHT increases COX-2 under normoxia but decreases COX-2 under hypoxia. DHT blunts HIF-1a following hypoxia (AR-independent).	Zuloaga & Gonzales [40]
Inflammation	Primary human brain VSMC	DHT	Anti-inflammatory effect of DHT (reduction of COX-2) is mediated by ER-beta, likely via conversion to 3β-diol	Zuloaga et al. [22]
Inflammation	Adult male Fisher 344 rats (intact & GDX)	T (in vivo) or ex vivo T treatment	T increases LPS-induced CBV inflammation (COX-2, iNOS, PGE2).	Razmara et al. [41]
EC senescence	Adult male SAMP8, control SAMR 1 mice	DHT	DHT treatment decreases hippocampal endothelial cell senescence.	Ota et al. [42]


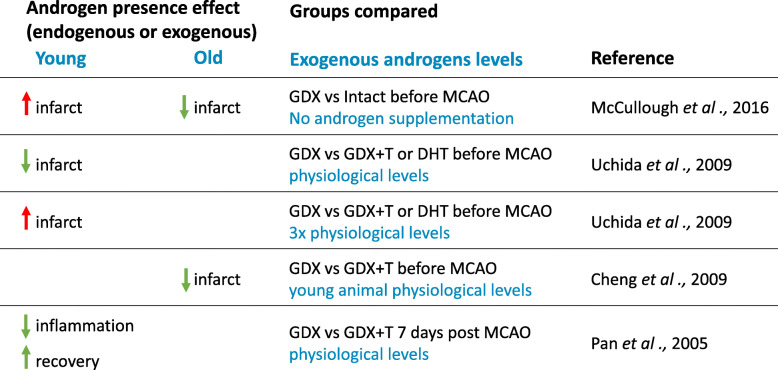

**Table 2:**
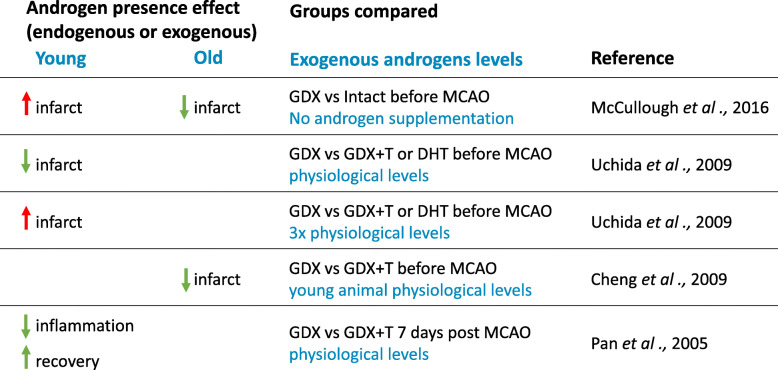
Androgens’ effects on outcomes of the MCAO model of stroke in male rodents

This table summarizes the outcomes of androgens’ modifications (removal by GDX or supplementation after GDX) on the MCAO model of stroke in young (< 6 months) versus old (12+ mo) rodents. Positive effects are indicated in green and negative effects are in red. GDX: gonadectomy; DHT: 5α-dihydrotestosterone; T: testosterone; MCAO: middle cerebral artery occlusion

## Effects of androgens on the cerebral vasculature

### Angiogenesis

Angiogenesis is the process of new blood vessel formation, requiring the migration and proliferation of endothelial progenitor cells (EPCs) or resident ECs. The majority of evidence shows that androgens promote angiogenesis, although most of this evidence comes from other vascular beds, while effects in the brain are less well studied. In male human EPCs (derived from the blood), androgen treatment in vitro stimulates EPC proliferation, migration, and colony formation through an AR-mediated pathway [29]. DHT significantly increases the proliferative, migratory, and adhesive abilities of male human blood-derived EPCs in a dose- and time-dependent manner and upregulates the levels of vascular endothelial growth factor (VEGF), a signal that stimulates the formation of the blood vessels [30]. Furthermore, DHT can modulate the human EPC proliferation and adhesion via a PI3-K/Akt pathway; however, these effects are dose-dependent and are diminished with low- or high-androgen doses [31]. Finally, the castration of adult rats results in decreased circulating EPC numbers, though T replacement did not restore their numbers [32]. Fadini and colleagues (2009) also showed that androgen replacement was able to only partially restore T levels, and the authors also suggest the effects of castration may be mediated by an estrogen-dependent pathway [32].

Studies on the effects of androgens on angiogenesis from brain ECs are lacking. Using in vitro assays comparing angiogenic potency in peripheral ECs of male AR knockout versus wild type mice, androgen signaling, via AR, has been shown to have a positive influence on angiogenesis [43]. In vivo, AR knockout mice also show impaired blood flow recovery with reduced capillary density in surviving skeletal muscle after hind-limb ischemia [43]. Androgens could have a sex-specific effect on angiogenesis in the peripheral vasculature, as males (but not females) show vascularization of matrigel plugs harvested from mice 14 days after gonadectomy and DHT treatment [44]. Conversely, a study in female songbird brains showed that T increased vascularization and expression of pro-angiogenic growth factors, including VEGF and its high-affinity receptor, VEGF-R2 [33]. Although this single study in the songbird brain and many studies in other vascular beds suggest a pro-angiogenic role of androgens, studies examining the effects of androgens on angiogenesis in the human and rodent brain specifically are lacking.

Androgen-dependent regulation of angiogenesis may be altered with aging. Indeed, when treated with DHT, fibroblasts isolated from young men increase VEGF production, while fibroblasts isolated from older men were not responsive to DHT [45]. Human umbilical vein ECs (HUVECs) treated with conditional media from these fibroblasts displayed increased angiogenic potential. These effects were AR dependent, as they were blocked by flutamide (AR antagonist). By contrast, this effect was lost when cells were isolated from older men, due to an impairment of AR translocation into the nucleus [45]. This study highlights the importance of age in androgen effects and supports the hypothesis that the same levels of androgens may be less effective in older versus younger men.

### Cerebrovascular reactivity

The brain’s vasculature responds and adapts to energy/oxygen demands via neurovascular coupling. ECs, pericytes, astrocytes, neurons, and the extracellular matrix form the neurovascular unit, which allows for focal rapid increases in CBF in response to neuronal activity. Moreover, CBF is maintained relatively constant within a range of systemic blood pressures due to cerebrovascular autoregulation. Indeed, VSM cells have the ability to constrict when intravascular pressure increases, therefore adjusting the vascular tone in response to changes in the arterial pressure [8]. In the cerebral vasculature, as in any other vascular bed, the blood flow is regulated by the delicate balance of vasoconstrictors, which induce smooth muscle contraction (e.g., endothelin-1, thromboxane), and vasodilators that cause relaxation [e.g., nitric oxide (NO), prostacyclin, and endothelial-derived hyperpolarizing factors (EDHF)].

Chronic androgen exposure has been shown to regulate the vascular tone through their influence on both vasoconstrictor and vasodilator pathways. In rodents, multiple studies have shown that chronic exposure to androgens promotes vasoconstriction of cerebral vessels. This is supported by findings that castration in male rats causes vasodilation of the middle cerebral arteries (MCA) [34–36]. Indeed, MCA wall thickness and myogenic tone were significantly greater in arteries from intact and T-treated rats compared to castrated rats 4 weeks after castration. Chronic T treatment of MCA segments ex vivo did not alter endothelin-1 (ET-1, vasoconstrictor) nor prostacyclin (PGI2, vasodilator) production, suggesting androgen’s effects are not mediated by these pathways [35]. However, other potential pathways for androgens’ regulation of the vascular tone were identified in a subsequent study. It was found that T treatment enhances thromboxane-A2, a potent vasoconstrictor and prothrombotic factor, in rat cerebral blood vessels [36]. Furthermore, both of these studies found that T affects myogenic tone through modulation of EDH, but independently of NO and cyclooxygenase-1 (COX-1) pathways [35, 36]. These studies were performed in males, while the effects of androgens on vascular reactivity in the female cerebral vasculature are yet to be determined.

It is noteworthy that acute androgen treatment has been shown to cause vasorelaxation in a variety of other vascular beds such as coronary, mesenteric, and prostate arteries. These studies show that this effect is non-genomic, AR-independent, and is likely mediated by calcium-activated potassium channels (BKCa) [46–49]. Acute androgen treatment has yet to be tested in the rodent cerebral circulation; however, BKCa-dependent relaxation has been described in rat cerebral arteries and could potentially be engaged by androgens [50]. Of note, T induces relaxation of the male canine basilar artery in vitro by blockage of voltage-dependent Ca^2+^ channels and, to a lesser extent, by activation of K^+^ channels. This is a rapid, non-genomic effect independent of the AR or aromatase activity [51].

Androgens’ effects on cerebrovascular reactivity seem to differ depending on the dose and duration of treatment (acute vs. chronic). This highlights the need for deeper investigation into the interaction between androgens and the cerebral vasculature. Further, most of the studies regarding long-term T treatment did not assess its local metabolism into estrogens, which may be relevant since estrogens are vasodilators [19].

In humans, T seems to influence circulating levels of the powerful vasoconstrictor ET-1 [52]. An increase of plasma ET-1 levels is observed in individuals undergoing female-to-male gender transition that are treated with T [52]. Conversely, plasma ET-1 levels were reported to be higher in males with hypogonadism and seemed to decrease after T administration [53]. This suggests a sex-specific action of androgens on ET-1 levels in humans, with T decreasing ET-1 in men but increasing it in individuals undergoing female-to-male transition.

### Blood-brain barrier

The cerebral vasculature is unique in its barrier properties, allowing selective regulation of the movement of molecules and cells between the blood and the CNS [known as the blood-brain barrier (BBB)]. The BBB results from the selectivity of the tight junctions between ECs of the central nervous system vessels. The properties of the BBB are maintained by critical interactions with other cells in the NVU, such as astrocytes. Astrocytic end-feet surround the ECs of the BBB, forming the “glia limitans” and providing biochemical support. The BBB protects the brain tissue from toxins and pathogens and helps regulate its homeostasis. Alterations of the BBB properties are an important component of pathology and progression of different cerebrovascular diseases. For more details about the BBB, we refer the reader to the review by Daneman and Part [54].

There are some inconsistencies in the literature regarding the effects of androgens on the BBB under physiological conditions, but the majority of the evidence suggests they are protective in males. Their effects on the BBB in females are unknown. In male mice, chronic depletion of T (by castration) increases the permeability of the BBB in the medial preoptic area, a highly sensitive brain area with respect to gonadal T. These effects can be reversed by T replacement [37]. The increase in BBB permeability after depletion of T is due to loss of expression of tight junction proteins such as claudin-5 and ZO-1 [37]. Conversely, a recent study shows that T supplementation for 6 weeks to young adult male Wistar rats compromises the blood spinal cord barrier as shown by a reduced expression of tight junction proteins, such as occludin, Jam 1, and VE-cadherin in the spinal cord [38]. This indicates that androgens’ effects on the blood barrier could be region-specific. Interestingly, in other vascular beds, DHT treatment increases occludin expression and transendothelial electrical resistance (an indicator of permeability) in cultured HUVECs. Preincubation with an AR antibody inhibited this effect, suggesting it is mediated by the plasma membrane-associated receptor [55].

In contrast to the protective effects of androgens on the BBB under physiological conditions, under pathological conditions, such as in a stroke model, DHT has been shown to have deleterious effects. These effects include downregulation of claudin-5 and upregulation of the proinflammatory chemokine CCL2, which is known to induce BBB damage [56, 57]. Further investigation is thus needed to confirm the role of androgens under normal and more importantly pathological conditions.

### Cerebrovascular inflammation and oxidative stress

Considering its important roles in brain function, damage caused to the cerebral vasculature by inflammation and/or oxidative stress contributes significantly to the pathogenesis of cerebrovascular diseases. During cerebrovascular inflammation, cells release a variety of pro-inflammatory mediators such as cytokines, chemokines, and proinflammatory enzymes, such as cyclooxygenase-2 (COX-2) and inducible nitric oxide synthase (iNOS) leading to increased oxidative stress and subsequent cell damage [58]. Both COX-2 and iNOS are hence used as markers of cellular inflammation. Inflammation and oxidative stress create a feedback loop of processes that can cause injury to and dysfunction of the vasculature and breakdown of the BBB [59]. Furthermore, increased permeability of the BBB can allow greater infiltration of leukocytes into the brain, increasing inflammatory processes and exacerbating cerebrovascular diseases.

Androgens appear to have opposite effects on cerebrovascular inflammation under physiological versus pathophysiological conditions. Under basal conditions, pro-inflammatory effects are often reported. DHT, via AR, stimulates cerebrovascular inflammation by increasing COX-2 and iNOS levels in male cerebral arteries in vivo and ex vivo, as well as in the primary human brain VSM cells [39, 40]. Conversely, under pathological conditions of endotoxin-induced inflammation, hypoxia, or ischemia, DHT is often reported to have anti-inflammatory effects. Under hypoxic conditions, DHT decreases HIF-1α and COX-2 levels in castrated male cerebral arteries in vivo and ex vivo, as well as in primary human brain VSMs and ECs [23, 40]. Similar anti-inflammatory effects of DHT have been observed in peripheral vascular cells under pathological conditions (e.g., cytokines or LPS-induced inflammation, ischemia) in vitro [40, 60, 61]. In contrast to DHT, T appears to have pro-inflammatory effects in the presence of LPS. In rats, androgen depletion by castration does not affect cerebrovascular inflammation under basal or inflammatory conditions of an endotoxin-induced (LPS) inflammation model [41]. However, chronic in vivo T treatment (4 weeks prior to LPS injection) exacerbates cerebrovascular inflammation by significantly augmenting the expression of COX-2 and iNOS in the cerebral vessels of male rats after LPS injection. This effect was maintained ex vivo in pial arteries [41], suggesting that T has long-lasting effects on cerebrovascular inflammation. These contradictory effects of T and DHT may be mediated by different mechanisms of action.

In contrast to DHT’s pro-inflammatory effect under a physiological condition which is AR-dependent, under pathophysiological conditions DHT decreases vascular inflammation via an AR-independent mechanism by conversion to 3β-androstanediol and activating ERβ [22, 23, 40]. Thus, effects of androgens on cerebrovascular inflammation seem to vary by androgen type and under physiological versus pathological conditions.

Systemic inflammation increases with age and has been associated with cognitive decline [62, 63]. T deficiency accelerates neuronal and vascular aging of senescence-accelerated mouse prone 8 (SAMP8), a mouse model of aging, and these parameters were ameliorated by DHT treatment. SIRT1, a NAD-dependent deacetylase, played an important role in the protective effect of DHT against oxidative stress-induced endothelial senescence in the hippocampus of these mice [42]. Thus, DHT also seems to be protective against EC senescence in the brain’s vasculature.

Androgens are also reported to be potent antioxidants, as shown in ECs and VSMs from other vascular beds [64, 65]. DHT treatment of HUVECs protects them against oxidative stress (hydrogen peroxide)-induced apoptosis. The protective effect of DHT was associated with the inhibition of caspase-3, caspase-9, and p38 MAPK phosphorylation induced by reactive oxygen species [64]. Further studies are needed to determine if these antioxidant effects extend to the cerebral vasculature.

A main consequence of cerebrovascular inflammation and oxidative stress is atherosclerosis, which contributes directly to the pathogenesis of cerebrovascular diseases [66]. Athero/arterio-sclerosis can be caused by inflammation and fatty deposit accumulation (e.g., cholesterol, LDLs) in the vessel wall. Some studies show that androgens have detrimental effects on several atherogenic processes [36, 67]. Androgen treatment increases expression of atherosclerosis-related genes in cultured male human macrophages; however, this is not true for macrophages derived from females [67]. Contrastingly, in primary human brain VSM cells and in ex vivo rat cerebral arteries, DHT, via an AR-independent mechanism, decreases proinflammatory mediator pathways following chronic exposure to hypoxia with glucose deprivation [40]. Androgens, via AR, inhibit oxidative stress-induced platelet aggregation, reducing the risk of thrombosis [68]. Castrated rats show an increase in hydrogen peroxide-induced platelet aggregation. The addition of DHT at physiological doses significantly inhibited this aggregation, and the effect was blocked by flutamide [68]. These data illustrate a complicated role of androgens that could be beneficial on certain aspects but detrimental on others. Studies investigating the effects of androgens on cerebrovascular inflammation and oxidative stress in females are lacking.

### Sex differences

As mentioned above, the cerebral vasculature expresses sex steroid receptors and metabolizing enzymes; not surprisingly, sex differences in its structure and function have been identified. Indeed, sex differences in human regional CBF were detected 3 decades ago by Rodriguez et al., with women showing an 11% higher CBF than men across all regions examined [69]. Since then, other differences in cerebral vasculature function have been demonstrated [for a detailed review of sex differences in the cerebral vasculature, we refer the readers to Robison et al. 2019). For example, differences in response to ET-1 or angiotensin II (Ang II) stimulation ex vivo in human cerebral arteries have been described. When stimulated, cerebral arteries from both middle-aged men and women upregulate ET-1 receptors and Ang II production; however, arteries from women were significantly less responsive to both vasoconstrictors [70]. Sex differences have also been identified in mouse cerebral arteries in response to Ang II. Cerebral arteries isolated from young adult wild-type male mice showed greater contractile responses to Ang II and greater generation of reactive oxygen species than arteries from females, potentially leading to a greater oxidative stress in males [71]. A recent, very thorough, study described numerous sex differences in the structure and function of rat middle cerebral arteries [72]. Wall thickness and the inner diameter of the MCA were smaller in 3-month Sprague Dawley female rats compared to males. Further, vascular smooth muscle cells were fewer in female MCA and showed a weaker contractile capability in vitro. Female MCA also had a greater myogenic tone and wall stress but less distensibility. Finally, MCA of male and female rats displayed similar vasoconstrictive responses to increases in perfusion pressure; however, at high-pressure levels (140–180 mmHg), female vessels failed to constrict and exhibited forced dilation [72]. Taken together, these results demonstrate sex differences in the function of both human and rodent cerebral arteries.

## Cerebrovascular diseases

According to the World Health Organization, cerebrovascular disease (CBVD) is the second leading cause of death behind cardiovascular disease, accounting for more than 10% of all deaths worldwide [73]. CBVDs, mainly stroke and vascular contributions to cognitive impairment and dementia (VCID), are also leading causes of disability. Here, we review how androgens affect these diseases and interact with their risk factors (Fig. 2).

### Stroke

The deadliest CBVD is stroke, which kills about 140,000 Americans per year, making it the 5th leading cause of death in the USA [74]. A stroke is the result of an interruption or reduction in CBF, which can lead to oxygen and nutrient deprivation of the brain resulting in subsequent damage. In ~85% of cases, stroke is a consequence of the stenosis/occlusion of a blood vessel (ischemic stroke); stroke can also be caused by rupture of vessels or aneurysms (hemorrhagic stroke; 15%) [75]. Stroke incidence and mortality rates are highly dependent on age and hormonal status [76, 77]. While stroke incidence is reported to be higher in men than in women during childhood and early adulthood, this sex difference disappears in middle-aged individuals and is even reversed in the elderly age group. For extensive review on the effects of sex and sex hormones in stroke prevalence, pathology, and outcomes, please refer to Roy-O’Reilly et al. [78].

The influence of androgens on stroke risk varies with age, such that high levels increase risk in the young, but low levels increase risk in the elderly. An increased incidence of stroke is observed in male neonates and infants (< 4 years old) compared to females of the same age. This is likely attributable to the two postnatal surges in T levels that occur at 1 week and 4–6 months after birth in boys [79, 80]. In pediatric populations, elevated T levels are associated with 4–5 fold increased risk of stroke, independent of pubertal status [81, 82]. Furthermore, a positive correlation is noted between high T levels and stroke. Each 1 nmol/l increase in serum T in boys is correlated with a 1.3-fold increase in the odds of cerebral thromboembolism [81]. Conversely, low androgen levels are linked to increased stroke risk with aging. In elderly men, lower total T level predicts increased incidence of stroke and poorer functional outcomes after adjusting for conventional risk factors for cardiovascular disease [83–85]. Moreover, serum T levels are lower in male stroke patients and are correlated with stroke severity, mortality, and infarct size [77, 86]. Taken together, these studies suggest that androgens’ effects on stroke appear to be dose-dependent, with both too high and too low levels of androgens putting men at increased risk of stroke. T altering therapies have diverse effects in men. For example, men undergoing androgen deprivation therapy for prostate cancer are at higher risk of ischemic stroke [87]. However, T replacement therapy in males over 65 years old has also been shown to increase the risk of adverse cardiovascular and cerebrovascular events [88]. The small size and the unique population of this clinical trial prevent generalization of the adverse effects of T. Although most of these studies have focused on men, a recent study examined the relationship between CBVD and androgens in women. In middle-aged women (40–60 years old), free T levels positively correlated with the carotid plaque area and hence a greater CBVD incidence [89].

Moreover, the balance between T and E2 is also important for CBVD risk. Among older men (60–90 years old), extremely low or high T/E2 values are associated with an increased incidence of CBVD [90]. Further, among post-menopausal women, a higher risk for incidence of cardiovascular disease is associated with an elevated T/E2 ratio [91].

In line with clinical data showing that high-androgen levels increase stroke risk in younger populations, animal models demonstrate that young adult male rodents exhibit worse pathology and functional outcomes following cerebral ischemia compared to females [92–98]. These sex differences could be due to sex hormones or sex chromosomal complement. In order to tease the contributions of sex hormones versus sex chromosome complement, the four-core genotype (FCG) model is used. In this model, the sex-determining SRY gene is moved from the Y chromosome to an autosome, allowing the generation of XX males (XXM) and XY females (XYF) [99]. The generated XXM have a hormonal status comparable to XY males (the same is true for XYF and XX females); therefore, the comparison between the two allows a better understanding of the chromosome versus hormone effects. Using the middle cerebral artery occlusion (MCAO) stroke model in the FCG mice, Manwani et al. demonstrate that sex differences in ischemic stroke sensitivity appear to be shaped by sex hormones, rather than sex chromosomal complement. Indeed, young adult males (XXM and XYM) had significantly higher infarct volumes as compared with gonadal females [100]. In further support of the role of sex hormones, in young adult male rats, castration is protective against ischemic stroke injury, as evident by a reduced infarct volume after MCAO. Supplementation with T or DHT to physiological levels restores infarct volumes to levels seen in intact males [56, 101–104]. Moreover, the effects of androgens on ischemic injury outcomes appear to be dose-dependent. In young male mice (2 months), Uchida et al. compared infarct size of intact, GDX, and GDX animals treated with either a low (1.5 mg) or a high (5 mg) dose of T [101]. Castration and low-dose androgen treatment 1 week before MCAO reduced infarct size, whereas high-dose T treatment exacerbated damage. These androgen effects were AR-dependent, as they were mimicked by DHT and blocked by flutamide. Of note, low-dose T-treated mice showed better functional recovery than untreated GDX controls 3 days after MCAO. However, this benefit was no longer apparent in T-treated mice at 7 days post-stroke, suggesting a time-sensitive effect [101].

Infarct size varies by age and sex in mouse models of stroke. Compared to age-matched females, young (5–6 months) males had significantly higher infarct volumes; however, this sex difference is reversed in middle-aged (14–15 months) mice, and no sex difference is found in 20–22-month-old mice [96]. These sex differences are generally attributed to the protective roles of estrogens in young females. However, it was recently shown that despite the lack of effect of sex chromosome complement in young mice, in late middle-aged/aged mice (18–20 months), sex chromosomal complement may emerge as a major contributor as XX females and XX males have significantly larger infarct volumes 72 h post MCAO than XY mice of either sex, while no significant difference in hormone levels is detected among all aged FCG mice [105]. This increased infarct size in the XX mice was shown to be due to enhanced inflammation. Post-stroke inflammation, which leads to secondary neuronal damage, is hypothesized to be enhanced in old XX mice due to a failure of X-chromosome inactivation with age. Regardless of the mechanism in females, the infarct size is smaller in middle-aged and aged males compared to young males, which in mice is supported by data showing that androgens increase infarct size in young animals, as previously mentioned.

In rodent models, the effects of androgens appear to vary with aging. In contrast to the detrimental effects of T in young rodents, supplementing T in middle-aged rodents (12–14 months) to the physiological levels ordinarily seen in young males (3 months) 1 week before MCAO reduces infarct size [102]. The effects of T in both young (increase infarct) and middle-aged rodents (decrease infarct) seem to be AR-dependent, as they are blocked by flutamide [101, 102]. Furthermore, infarct sizes are comparable in middle-aged male aromatase knockout (ArKO) mice [102], suggesting that T is not acting via conversion to estradiol.

Androgen effects on stroke also vary by timing of androgen administration relative to stroke onset. Interestingly, as opposed to other studies where androgen modulation happened before or immediately after stroke, T replacement (physiological levels) improves functional recovery and reduces inflammation in the area of injury when administered 7 days post MCAO in young castrated rats [106]. This data suggests that androgens’ effects also depend on the time of administration.

Taken together, these observations highlight the complexity of androgens’ role in stroke, showing that their effects are sex-, age-, dose-, and even time-dependent (Table 2).

### Vascular contributions to cognitive impairment and dementia (VCID)

Maintenance of appropriate CBF and BBB function is crucial for the structural and functional integrity of the brain. Thus, it is not surprising that alterations in cerebral blood vessels have a profound impact on cognitive function. Vascular contributions to cognitive impairment and dementia (VCID) are a heterogeneous group of disorders defined by cognitive impairment (mild) or vascular dementia (VaD, in severe cases) resulting from cerebrovascular pathology. Diverse vascular alterations contribute to the pathogenesis of VCID, ranging from systemic conditions affecting global cerebral perfusion (such as cardiovascular disease, carotid artery stenosis/occlusion) or alterations involving cerebral blood vessels, most commonly small size arterioles or venules including arteriosclerosis, cerebral amyloid angiopathy (CAA), and cerebral autosomal dominant arteriopathy with subcortical infarcts and leukoencephalopathy (CADASIL). Of note, the stroke itself can lead to VCID, as one out of three stroke survivors will develop dementia [107]. For an in-depth review of VCID, we recommend Iadecola et al. 2013 [1]. VCID symptoms can include mental slowness and deficits in executive function and memory, as well as a variety of behavioral, psychological, and neurological disturbances. VaD is the second most common form of dementia behind Alzheimer’s disease (AD), accounting for ∼15% of dementia cases [108]. Moreover, VCID is comorbid in up to 80% of AD patients, resulting in multi-etiology dementia [109, 110].

VaD is slightly more common in men [111–115]; however, this trend seems to be reversed in the elderly population, as VaD is more prevalent among women above 85–90 years old [114, 116]. Data about the association between dementia and serum T levels is mixed [117–120]. In elderly men (71–93 years old), endogenous T levels were not associated with risk for cognitive decline and AD, whereas higher estrogen levels increased risk for cognitive decline and AD [119]. A more recent longitudinal study found that men (71–88 years old) with the lowest T levels had increased risk of developing dementia (unspecified etiological causes) compared to those in the highest quartile [120].

Surprisingly, androgens’ effects in animal models of VCID, such as the chronic cerebral hypoperfusion models (e.g., unilateral carotid artery occlusion or bilateral carotid artery stenosis) have yet to be assessed. However, in a rat model of post-stroke dementia, it was found that T replacement after castration increased cognitive deficits following stroke in middle-aged males [122]. It is unclear if the cognitive effect observed was dependent on infarct volume or was independent of stroke severity. However, this effect was found to be mediated by increased oxidative stress, as it could be inhibited by a free-radical scavenger. In contrast to the detrimental effects of T in the context of stroke, in sham animals, T actually improved cognitive performance [122]. Therefore, future studies are needed to determine the role of androgens in models of cerebral small vessel disease and chronic cerebral hypoperfusion.

### Non-vascular effects of androgens affecting CBVD outcomes

Even though it is beyond the scope of this review, it is important to mention that the role of androgens in CBVD’s goes beyond their effects on the vasculature itself. Androgens are also known to influence several neural cells, as well as other aspects of these diseases such as inflammation and oxidative stress outside of the vasculature. Indeed, protective effects of T are also found in studies of oxidative stress, β-amyloid toxicity, and serum deprivation in neurons [123–126]. Additionally, T has been shown to increase glutathione levels (an antioxidant) in cultured neuronal and glial cell lines [127]. In the MCAO stroke model, GDX rats treated with T for 10 days starting 24 h after induction of brain ischemia show increased antioxidant effects, as well as increased brain-derived neurotrophic factor (BDNF) levels and neurogenesis, associated with enhanced recovery [128]. Further, both androgens and the cerebral vasculature regulate several aspects of neural development that could affect disease outcomes, such as neurogenesis and myelination. Indeed, the vasculature is an important scaffold for neuronal cell migration [129] and oligodendrocytes [121]. During myelin repair, blood vessels guide implanted Schwann cell migration in the adult demyelinated mouse spinal cord [130]. T affects oligodendrocytes number during development via an AR-dependent pathway [131] and is also important for myelin repair [132]. Endothelial cells have also been shown to stimulate the self-renewal of neural stem cells by secreting several factors [133–135]. T also enhances neurogenesis in young adult rodents as evident by the increasing survival of newly formed neurons. This effect is not observed in females and middle-aged males [136–138]. Thus, androgens may affect CBVD through non-vascular mediated mechanisms.

## Risk factors

**Fig. 3:**
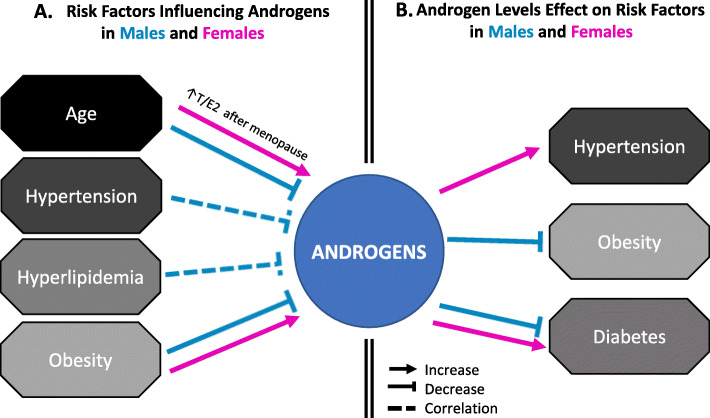
Interaction between androgens and cerebrovascular disease risk factors. a Schematic representation of risk factors influencing or correlating with androgen levels in males and females. b Schematic representation of how androgen levels affect/correlate with risk factors for cerebrovascular diseases. Relations are shown in pink in females, and those in males are shown in blue. T/E2, testosterone to estrogen ratio increases after menopause in females

Androgens’ effects on CBVD could be indirect by altering several risk factors. In this section, we will briefly discuss risk factors that affect and/or are affected by androgens (Fig. 3). For an extensive review of risk factors for VCID and the way they differ between sexes, we refer readers to Gannon et al. [139].

### Sex and age

Overall, men appear to be at a higher risk for CBVD throughout most of the lifespan, although in elderly individuals this trend is reversed [78, 116, 139]. This is believed to be a result of menopause, which causes an increase in the androgen to estrogen ratio in women. However, this could also be due in part to aging, as age is one of the major risk factors for CBVD, and women are known to benefit from a survival bias [140].

**Fig. 4:**
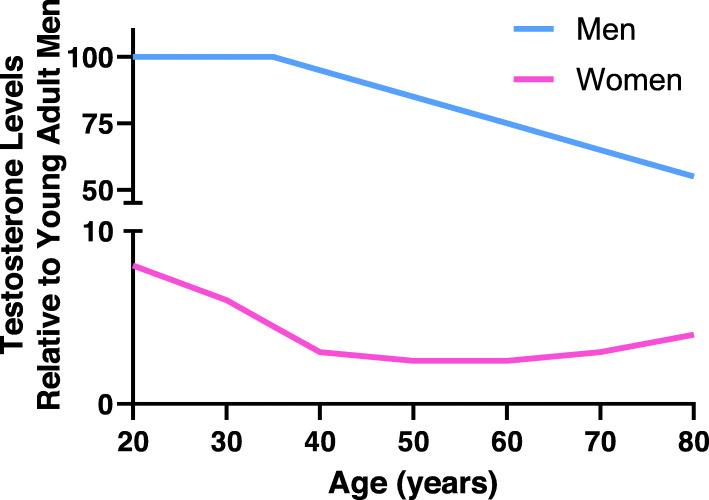
Schematic representation of testosterone levels during adulthood. Plasma testosterone levels (relative to those found in young men) are plotted during adulthood in men (blue) and women (pink)

Androgen levels decline with age in both men and women (Fig. 4). In men, beginning around the age of 35–40 years, circulating T levels decrease by approximately 1–3% per year [141]. Around 20% of men older than 60 years old and 50% of men older than 80 years old have serum T concentrations below the normal range for young men [62, 142].

Women, in general, have a 20- to 25-fold lower circulating concentration of androgens compared with men [143]. T levels also decline in women with age [144]. For example, in the fourth decade of life and prior to menopause, T levels approach 50% of those seen in the third decade of life [145]. Upon completion of menopause, T levels are ~15% of premenopausal levels [62, 145]. However, one study reported increased T levels in women 20–30 years post-menopause compared to women less than 20 years post-menopause [146] (Fig. 4).

This decline in T levels with age in both men and women may be linked to sex-specific effects on peripheral (non-cerebral) vascular aging (arterial stiffening and endothelial dysfunction). Sex differences in vascular aging in response to androgens, specifically T are reviewed by Moreau et al. [147].

Of note, women with polycystic ovary syndrome (PCOS) have elevated levels of plasma androgens, which are associated with increased risk of cardiovascular disease including stroke [148, 149]. A small study involving 18 women with PCOS showed alterations of white matter microstructure and compromised cognitive performance (indicators of VCID) compared to controls, independent of age, education, and body mass index (BMI) [150].

### Hypertension

Hypertension (high blood pressure) is known to increase risk of VCID [112, 151]. Men are more prone to hypertension than women throughout most of the lifespan, although this sex difference dissipates during aging and is even reversed after menopause [152]. Hypertensive men have significantly lower T levels than non-hypertensives, even after adjusting for age and body mass index. In support of this, both systolic and diastolic blood pressure (BP) are inversely correlated with T levels in adult men [153]. Conversely, in women, a positive association between T and BP is observed, even after menopause [154]. Further, T supplementation in female-to-male transgender individuals leads to a significant increase in BP [155]. Thus, the relationship of androgens with blood pressure is sex specific, with high blood pressure being linked to low androgen levels in men, but high androgen levels in women.

### Hyperlipidemia

Hyperlipidemia is defined by elevated total cholesterol (TC), low levels of high-density lipoprotein cholesterol (HDL), and high levels of low-density lipoprotein cholesterol (LDL). In the US, low HDL prevalence is 3-fold higher among adult men than women [156]. Total cholesterol levels are similar between men and women in the young adult (20-39 yo) and middle aged (40-59 yo) groups. However, for adults aged 60 and over, there is 2.5× higher prevalence of high total cholesterol in women [156]. Hyperlipidemia is associated with increased risk of cardiovascular disease. In men, but not in women, low HDL levels and high triglyceride levels are associated with increased all-cause dementia risk [157]. In men, several cross-sectional and prospective observational studies show that low endogenous T is associated with high LDL and low HDL. Exogenous T administration is associated with decreased HDL but also with beneficial decreases in LDL and TC [158, 159]. Low androgen levels are therefore associated with hyperlipidemia and, by consequence, higher CBVD risk in men. It is noteworthy that in female-to-male transgender individuals, T treatment leads to decreased HDL levels, as well as higher LDL and triglycerides levels [160, 161]. Despite this change in lipid profile, as well as an increase in blood pressure (mainly systolic), meta-analyses and retrospective studies do not report any increase in CBVD risk among female-to-male transgender individuals [162].

### Diabetes

Diabetes, both type 1 and type 2, increases risk for CBVD. Type 1 diabetic (T1D) patients suffer from a shortened lifespan (∼68 years in women, ∼66 years in men) [163], which is probably the reason why the role of type 1 diabetes in VaD or VCID risk has yet to be determined. However, stroke risk is increased 3.7-fold for males and 4.8-fold for females with T1D compared to matched controls [164, 165].

Type 2 diabetes (T2D) increases risk of cognitive dysfunction and doubles the risk of VaD [166–168]. Type 2 diabetics have worse cognitive performance than those with normal blood glucose levels. Further, diabetes duration of 15 years or more is associated with significantly poorer processing speed and executive function [166].

There are sex differences in the effect of T2D on VaD. Using a pooled metanalysis study that included 2.3 million people, Chatterjee et al. showed that individuals with T2D are at ∼60% greater risk for the development of all-cause dementia, and VaD in particular. When this data was stratified by sex, it showed that diabetic women have a 19% greater risk for the development of VaD than diabetic men. After adjusting for possible confounders, women with diabetes had a 120% greater risk for the development of VaD compared to non-diabetic women, whereas in men, diabetes increased the risk by 70% compared to non-diabetic men [169]. In support of this, among VaD patients, diabetes is more prevalent in women than men (64% vs. 36%) [170].These results suggest that diabetes is a greater risk factor for VCID in women compared to men.

Men are more prone to develop T2D than women of a similar body weight, and even at a younger age. This may be linked to men usually having more visceral fat and higher degree of insulin resistance compared to women of comparable age and BMI [171]. T deficiency is linked to an increased risk of T2D in men [158], with higher T level being associated with a decrease in T2D risk [172]. Additionally, an interaction between diabetes and low androgen levels in men is shown to increase inflammation, oxidative stress, and atherosclerotic markers leading to higher risk of cardiovascular events [173]. These findings highlight a possibly protective role of androgens in diabetic men. Conversely, in women, androgen excess predisposes to insulin resistance and T2D [174]. This is of relevance in the case of PCOS, where women have higher incidence of insulin resistance and a higher risk of dementia such as AD [175]. Thus, the effects of androgens on metabolic disease appear to be sex-specific.

Prediabetes, defined as glucose intolerance or insulin resistance in the absence of hyperglycemia, has also been found to be a risk factor for vascular dementia, all-cause dementia, and AD [176]. Men with impaired glucose tolerance (prediabetes) have significantly lower total testosterone levels. Further, total T is inversely associated with both impaired glucose tolerance and T2D in men, suggesting a protective role of androgens in metabolic disease [143]. Conversely, women with impaired glucose tolerance have significantly higher bioavailable T and E2 levels than those with normal glucose tolerance. Their T and E2 levels were positively associated with fasting plasma glucose [143]. Thus, androgens appear to have opposite associations with glucose tolerance in each sex, with low T being detrimental in men but beneficial in women.

### Obesity

Obesity can contribute to CBVD risk directly or indirectly by influencing other risk factors. Indeed, visceral adiposity (reflected by a high waist circumference, waist-to-hip ratio, or waist-to-height ratio) is associated with high stroke risk [177]. Visceral fat, but not subcutaneous fat, is also associated with cerebral small vessel disease, as reflected by higher incidence of white matter hyperintensities in individuals with high visceral fat [178]. Furthermore, midlife (40–45 years old) obesity increases risk of VaD, and individuals who are overweight have a 2× higher risk of VaD independent of cardiovascular disease, stroke, and diabetes comorbidities [179]. Additionally, lifestyle factors such as poor nutrition and lack of physical activity can contribute to both obesity and dementia risk (reviewed in [180, 181]).

In the USA, women are more prone to obesity than men [182]. In women, serum androgen levels have been shown to increase with increasing body mass index in post-menopausal, but not pre-menopausal women [183]. The link between obesity and androgens in women is reviewed in Pasquali et al. [184]. Interestingly, we have found that obese middle-aged female mice develop more severe glucose intolerance than obese males when on a high fat diet [185].

In men, there is a bidirectional relationship between obesity and T levels [186], whereby low androgen levels contribute to obesity and obesity decreases androgen levels. Specifically, decreases in androgens are associated with increased body mass index and obesity in men [187, 188]. In obese individuals, the increased aromatase activity/estrogen synthesis from fat mass leads to a reduction in T production by suppressing the hypothalamic-pituitary-testis pathway [189]. Further, low T, resulting from androgen deprivation therapy in prostate cancer patients, leads to an increase in visceral and subcutaneous abdominal fat [190]. This association of low androgen levels with obesity appears to be sex specific, with low androgen levels observed in obese men, but high androgen levels in overweight women.

All of these risk factors (hypertension, dyslipidemia, insulin resistance/type 2 diabetes, obesity) are key features of the metabolic syndrome, which is a known risk factor for CBVD and has been associated with lower androgen levels/hypogonadism in men. Lifestyle factors (such as diet and exercise) and T treatment may have additive beneficial effects of battling metabolic syndrome and its downstream complications on CBVD [186, 191–193].

## Conclusions

In summary, men are more prone to CBVD, including stroke and VCID, throughout most of their lifespan, although this sex differences dissipates and even reverses in the elderly. Sex differences in these diseases might be due to sex hormones such as androgens. Androgens’ effects on blood vessels have been studied in the scope of cardiovascular diseases, including stroke, which show that T effects on stroke vary with age and dosage in men. In the cerebrovasculature, both ECs and VSMs express androgen receptors and metabolizing enzymes, making the cerebral vasculature a direct target of androgens. Indeed, androgens affect several aspects of the cerebral vasculature such as angiogenesis, cerebrovascular reactivity, and BBB integrity (Table 1). However, most studies of androgen effects on blood vessels were done in vitro on isolated cells or in other vascular beds. Studies examining specific effects of androgens on the cerebral vasculature are sparse and virtually non-existent in females. Further, there is little to no information on how androgens regulate some aspects of vascular physiology such as pericyte or lymphatic function. Studies of androgens’ effects on VCID models are also lacking.

The important interactions between androgens, the cerebral vasculature, and several aspects of brain homeostasis affected in health and disease draw attention to hormone altering therapies such as androgen deprivation for prostate cancer and male-to-female transgender individuals and androgen replacement therapies in cases of hypogonadism, anabolic steroid use, and female-to-male transgender individuals. Particularly, as the population ages, T deficiency and hypogonadism are becoming increasingly recognized as a problem with negative consequences in men. As a result, androgen replacement is being investigated in various clinical modalities in men to reduce these negative effects of hypogonadism [194]. Androgen replacement therapy gains even more importance in the scope of CBVD risk factors such as hypertension, diabetes, and obesity/dyslipidemia, which have all been linked to low T levels. These same risk factors have also been seen in men with prostate cancer who have been treated with androgen deprivation therapy. Further studies evaluating the benefits/risks of hormone replacement therapy in the scope of aging and other risk factors are needed for both men and women.

The effects of sex hormones, notably androgens, vary based on dose, age, and disease state, highlighting the need for animal studies that accurately model clinical conditions (e.g., aged subjects, presence of common co-morbidities). Unfortunately, most studies reviewed here utilized young; otherwise, healthy male animals highlighting the need of more comprehensive animal study designs that take into consideration possible interactions between sex, age, and other risk factors that commonly occur with CBVD. Greater understanding of androgens’ effects on cerebrovascular function and disease will assist in developing personalized approaches and help determine the optimum dose and timing of androgen altering therapies.

## Data Availability

Not applicable.

## Abbreviations

AD: Alzheimer’s disease
Ang II: Angiotensin II
AR: Androgen receptor
ArKO: Aromatase knockout
BBB: Blood-brain barrier
BDNF: Brain-derived neurotrophic factor
BKCa: Calcium-activated potassium channels
BMI: Body mass index
CAA: Cerebral amyloid angiopathy
CADASIL: Cerebral autosomal dominant arteriopathy with subcortical infarcts and leukoencephalopathy
CBF: Cerebral blood flow
CBVD: Cerebrovascular disease
CNS: Central nervous system
COX-1: Cyclooxygenase 1
COX-2: Cyclooxygenase 2
DHT: 5α-Dihydrotestosterone
EC: Endothelial cell
EDHF: Endothelium-derived hyperpolarizing factors
EPC: Endothelial progenitor cell
ER: Estrogen receptor
ET-1: Endothelin1
FCG: Four-core genotype
GDX: Gonadectomy
GPER1: G-protein-coupled estrogen receptor
HDL: High-density lipoprotein cholesterol
HSD: 3β-Hydroxysteroid dehydrogenase
HUVEC: Human umbilical vein endothelial cell
iNOS: Inducible nitric oxide synthase
LDL: Low-density lipoprotein cholesterol
LPS: Endotoxin lipopolysaccharide
MCA: Middle cerebral arteries
MCAO: Middle cerebral artery occlusion
mER: Membrane-bound estrogen receptor
NO: Nitric oxide
NVU: Neurovascular unit
PCOS: Polycystic ovarian syndrome
SRT: 1 Sirtuin 1
T: Testosterone
T1D: Type 1 diabetes
T2D: Type 2 diabetes
TC: Total cholesterol
VaD: Vascular dementia
VCID: Vascular contributions to cognitive impairment and dementia
VEGF: Vascular endothelial growth factor
VSM: Vascular smooth muscle cell
XXM: XX males of the four-core genotype model
XYF: XY females of the four-core genotype model

## References

[1] Iadecola C (2013). The pathobiology of vascular dementia. Neuron..

[2] Armulik A, Genove G, Mae M, Nisancioglu MH, Wallgard E, Niaudet C (2010). Pericytes regulate the blood-brain barrier. Nature..

[3] Quaegebeur A, Lange C, Carmeliet P (2011). The neurovascular link in health and disease: molecular mechanisms and therapeutic implications. Neuron..

[4] Dyrna F, Hanske S, Krueger M, Bechmann I (2013). The blood-brain barrier. J NeuroImmune Pharmacol.

[5] Carare RO, Teeling JL, Hawkes CA, Puntener U, Weller RO, Nicoll JA (2013). Immune complex formation impairs the elimination of solutes from the brain: implications for immunotherapy in Alzheimer's disease. Acta Neuropathol Commun.

[6] Iliff JJ, Lee H, Yu M, Feng T, Logan J, Nedergaard M (2013). Brain-wide pathway for waste clearance captured by contrast-enhanced MRI. J Clin Invest.

[7] Laman JD, Weller RO (2013). Drainage of cells and soluble antigen from the CNS to regional lymph nodes. J NeuroImmune Pharmacol.

[8] Cipolla MJ. The cerebral circulation. integrated systems physiology: from molecule to function. San Rafael (CA)2009.

[9] Handa RJ, Ogawa S, Wang JM, Herbison AE. Roles for oestrogen receptor β in adult brain function. J Neuroendocrinol. 2012;24(1):160–73. 10.1111/j.1365-2826.2011.02206.x. 10.1111/j.1365-2826.2011.02206.x PMC3348521 21851428

[10] Watson PA, Chen YF, Balbas MD, Wongvipat J, Socci ND, Viale A (2010). Constitutively active androgen receptor splice variants expressed in castration-resistant prostate cancer require full-length androgen receptor. Proc Natl Acad Sci U S A.

[11] Chamberlain NL, Driver ED, Miesfeld RL (1994). The length and location of CAG trinucleotide repeats in the androgen receptor N-terminal domain affect transactivation function. Nucleic Acids Res.

[12] Tirabassi G, Delli Muti N, Corona G, Maggi M, Balercia G (2013). Androgen receptor gene CAG repeat polymorphism regulates the metabolic effects of testosterone replacement therapy in male postsurgical hypogonadotropic hypogonadism. Int J Endocrinol.

[13] Lucas-Herald AK, Alves-Lopes R, Montezano AC, Ahmed SF, Touyz RM (2017). Genomic and non-genomic effects of androgens in the cardiovascular system: clinical implications. Clin Sci (Lond).

[14] Weiser MJ, Foradori CD, Handa RJ (2008). Estrogen receptor beta in the brain: from form to function. Brain Res Rev.

[15] Guo X, Razandi M, Pedram A, Kassab G, Levin ER (2005). Estrogen induces vascular wall dilation: mediation through kinase signaling to nitric oxide and estrogen receptors alpha and beta. J Biol Chem.

[16] Levin ER (2009). G protein-coupled receptor 30: estrogen receptor or collaborator?. Endocrinology..

[17] Mizukami Y (2010). In vivo functions of GPR30/GPER-1, a membrane receptor for estrogen: from discovery to functions in vivo. Endocr J.

[18] Pabbidi MR, Kuppusamy M, Didion SP, Sanapureddy P, Reed JT, Sontakke SP (2018). Sex differences in the vascular function and related mechanisms: role of 17beta-estradiol. Am J Phys Heart Circ Phys.

[19] Robison LS, Gannon OJ, Salinero AE, Zuloaga KL (2019). Contributions of sex to cerebrovascular function and pathology. Brain Res.

[20] Borst SE, Shuster JJ, Zou B, Ye F, Jia H, Wokhlu A (2014). Cardiovascular risks and elevation of serum DHT vary by route of testosterone administration: a systematic review and meta-analysis. BMC Med.

[21] Cai JJ, Wen J, Jiang WH, Lin J, Hong Y, Zhu YS (2016). Androgen actions on endothelium functions and cardiovascular diseases. J Geriatr Cardiol.

[22] Zuloaga KL, Swift SN, Gonzales RJ, Wu TJ, Handa RJ (2012). The androgen metabolite, 5alpha-androstane-3beta,17beta-diol, decreases cytokine-induced cyclooxygenase-2, vascular cell adhesion molecule-1 expression, and P-glycoprotein expression in male human brain microvascular endothelial cells. Endocrinology..

[23] Zuloaga KL, O'Connor DT, Handa RJ, Gonzales RJ (2012). Estrogen receptor beta dependent attenuation of cytokine-induced cyclooxygenase-2 by androgens in human brain vascular smooth muscle cells and rat mesenteric arteries. Steroids..

[24] Broughton BR, Brait VH, Guida E, Lee S, Arumugam TV, Gardiner-Mann CV (2013). Stroke increases G protein-coupled estrogen receptor expression in the brain of male but not female mice. Neurosignals..

[25] Evanson KW, Goldsmith JA, Ghosh P, Delp MD (2018). The G protein-coupled estrogen receptor agonist, G-1, attenuates BK channel activation in cerebral arterial smooth muscle cells. Pharmacol Res Perspect.

[26] Gonzales RJ, Ansar S, Duckles SP, Krause DN (2007). Androgenic/estrogenic balance in the male rat cerebral circulation: metabolic enzymes and sex steroid receptors. J Cereb Blood Flow Metab.

[27] Shih HC, Lin CL, Wu SC, Kwan AL, Hong YR, Howng SL (2008). Upregulation of estrogen receptor alpha and mediation of 17beta-estradiol vasoprotective effects via estrogen receptor alpha in basilar arteries in rats after experimental subarachnoid hemorrhage. J Neurosurg.

[28] Zuloaga KL, Davis CM, Zhang W, Alkayed NJ (2014). Role of aromatase in sex-specific cerebrovascular endothelial function in mice. Am J Phys Heart Circ Phys.

[29] Foresta C, Zuccarello D, De Toni L, Garolla A, Caretta N, Ferlin A (2008). Androgens stimulate endothelial progenitor cells through an androgen receptor-mediated pathway. Clin Endocrinol.

[30] Zhang H, Shi L, Ren GQ, Sun WW, Wang YB, Chen YK (2016). Dihydrotestosterone modulates endothelial progenitor cell function via RhoA/ROCK pathway. Am J Transl Res.

[31] Liu R, Ding L, Yu MH, Wang HQ, Li WC, Cao Z (2014). Effects of dihydrotestosterone on adhesion and proliferation via PI3-K/Akt signaling in endothelial progenitor cells. Endocrine..

[32] Fadini GP, Albiero M, Cignarella A, Bolego C, Pinna C, Boscaro E (2009). Effects of androgens on endothelial progenitor cells in vitro and in vivo. Clin Sci (Lond).

[33] Louissaint A, Rao S, Leventhal C, Goldman SA (2002). Coordinated interaction of neurogenesis and angiogenesis in the adult songbird brain. Neuron..

[34] Geary GG, Krause DN, Duckles SP (2000). Gonadal hormones affect diameter of male rat cerebral arteries through endothelium-dependent mechanisms. Am J Phys Heart Circ Phys.

[35] Gonzales RJ, Krause DN, Duckles SP (2004). Testosterone suppresses endothelium-dependent dilation of rat middle cerebral arteries. Am J Phys Heart Circ Phys.

[36] Gonzales RJ, Ghaffari AA, Duckles SP, Krause DN (2005). Testosterone treatment increases thromboxane function in rat cerebral arteries. Am J Phys Heart Circ Phys.

[37] Atallah A, Mhaouty-Kodja S, Grange-Messent V (2017). Chronic depletion of gonadal testosterone leads to blood-brain barrier dysfunction and inflammation in male mice. J Cereb Blood Flow Metab.

[38] Nierwinska K, Nowacka-Chmielewska M, Bernacki J, Jagsz S, Chalimoniuk M, Langfort J (2019). The effect of endurance training and testosterone supplementation on the expression of blood spinal cord barrier proteins in rats. PLoS One.

[39] Gonzales RJ, Duckles SP, Krause DN (2009). Dihydrotestosterone stimulates cerebrovascular inflammation through NFkappaB, modulating contractile function. J Cereb Blood Flow Metab.

[40] Zuloaga KL, Gonzales RJ (2011). Dihydrotestosterone attenuates hypoxia inducible factor-1alpha and cyclooxygenase-2 in cerebral arteries during hypoxia or hypoxia with glucose deprivation. Am J Phys Heart Circ Phys.

[41] Razmara A, Krause DN, Duckles SP (2005). Testosterone augments endotoxin-mediated cerebrovascular inflammation in male rats. Am J Phys Heart Circ Phys.

[42] Ota H, Akishita M, Akiyoshi T, Kahyo T, Setou M, Ogawa S (2012). Testosterone deficiency accelerates neuronal and vascular aging of SAMP8 mice: protective role of eNOS and SIRT1. PLoS One.

[43] Yoshida S, Aihara K, Ikeda Y, Sumitomo-Ueda Y, Uemoto R, Ishikawa K (2013). Androgen receptor promotes sex-independent angiogenesis in response to ischemia and is required for activation of vascular endothelial growth factor receptor signaling. Circulation..

[44] Sieveking DP, Lim P, Chow RW, Dunn LL, Bao S, McGrath KC (2010). A sex-specific role for androgens in angiogenesis. J Exp Med.

[45] Lecce L, Lam YT, Lindsay LA, Yuen SC, Simpson PJ, Handelsman DJ (2014). Aging impairs VEGF-mediated, androgen-dependent regulation of angiogenesis. Mol Endocrinol.

[46] Deenadayalu VP, White RE, Stallone JN, Gao X, Garcia AJ (2001). Testosterone relaxes coronary arteries by opening the large-conductance, calcium-activated potassium channel. Am J Phys Heart Circ Phys.

[47] Jones RD, Pugh PJ, Hall J, Channer KS, Jones TH (2003). Altered circulating hormone levels, endothelial function and vascular reactivity in the testicular feminised mouse. Eur J Endocrinol.

[48] Tep-areenan P, Kendall DA, Randall MD (2002). Testosterone-induced vasorelaxation in the rat mesenteric arterial bed is mediated predominantly via potassium channels. Br J Pharmacol.

[49] Yue P, Chatterjee K, Beale C, Poole-Wilson PA, Collins P (1995). Testosterone relaxes rabbit coronary arteries and aorta. Circulation..

[50] Nelson MT, Cheng H, Rubart M, Santana LF, Bonev AD, Knot HJ (1995). Relaxation of arterial smooth muscle by calcium sparks. Science..

[51] Ramirez-Rosas MB, Cobos-Puc LE, Munoz-Islas E, Gonzalez-Hernandez A, Sanchez-Lopez A, Villalon CM (2011). Pharmacological evidence that Ca(2)+ channels and, to a lesser extent, K+ channels mediate the relaxation of testosterone in the canine basilar artery. Steroids..

[52] Polderman KH, Stehouwer CD, van Kamp GJ, Dekker GA, Verheugt FW, Gooren LJ (1993). Influence of sex hormones on plasma endothelin levels. Ann Intern Med.

[53] Kumanov P, Tomova A, Kirilov G (2007). Testosterone replacement therapy in male hypogonadism is not associated with increase of endothelin-1 levels. Int J Androl.

[54] Daneman R, Prat A (2015). The blood-brain barrier. Cold Spring Harb Perspect Biol.

[55] Sumanasekera WK, Sumanasekera GU, Mattingly KA, Dougherty SM, Keynton RS, Klinge CM (2007). Estradiol and dihydrotestosterone regulate endothelial cell barrier function after hypergravity-induced alterations in MAPK activity. Am J Phys Cell Physiol.

[56] Cheng J, Alkayed NJ, Hurn PD (2007). Deleterious effects of dihydrotestosterone on cerebral ischemic injury. J Cereb Blood Flow Metab.

[57] Dimitrijevic OB, Stamatovic SM, Keep RF, Andjelkovic AV (2006). Effects of the chemokine CCL2 on blood-brain barrier permeability during ischemia-reperfusion injury. J Cereb Blood Flow Metab.

[58] Dauphinee SM, Karsan A (2006). Lipopolysaccharide signaling in endothelial cells. Lab Investig.

[59] Chrissobolis S, Miller AA, Drummond GR, Kemp-Harper BK, Sobey CG (2011). Oxidative stress and endothelial dysfunction in cerebrovascular disease. Front Biosci (Landmark Ed).

[60] Norata GD, Grigore L, Raselli S, Seccomandi PM, Hamsten A, Maggi FM (2006). Triglyceride-rich lipoproteins from hypertriglyceridemic subjects induce a pro-inflammatory response in the endothelium: molecular mechanisms and gene expression studies. J Mol Cell Cardiol.

[61] Osterlund KL, Handa RJ, Gonzales RJ (2010). Dihydrotestosterone alters cyclooxygenase-2 levels in human coronary artery smooth muscle cells. Am J Phys Endocrinol Metab.

[62] Horstman AM, Dillon EL, Urban RJ, Sheffield-Moore M (2012). The role of androgens and estrogens on healthy aging and longevity. J Gerontol A Biol Sci Med Sci.

[63] Lin T, Liu GA, Perez E, Rainer RD, Febo M, Cruz-Almeida Y (2018). Systemic inflammation mediates age-related cognitive deficits. Front Aging Neurosci.

[64] Xu ZR, Hu L, Cheng LF, Qian Y, Yang YM (2010). Dihydrotestosterone protects human vascular endothelial cells from H(2)O(2)-induced apoptosis through inhibition of caspase-3, caspase-9 and p38 MAPK. Eur J Pharmacol.

[65] Tam NN, Gao Y, Leung YK, Ho SM (2003). Androgenic regulation of oxidative stress in the rat prostate: involvement of NAD(P)H oxidases and antioxidant defense machinery during prostatic involution and regrowth. Am J Pathol.

[66] Zalba G, Fortuno A, San Jose G, Moreno MU, Beloqui O, Diez J (2007). Oxidative stress, endothelial dysfunction and cerebrovascular disease. Cerebrovasc Dis.

[67] Ng MK, Quinn CM, McCrohon JA, Nakhla S, Jessup W, Handelsman DJ (2003). Androgens up-regulate atherosclerosis-related genes in macrophages from males but not females: molecular insights into gender differences in atherosclerosis. J Am Coll Cardiol.

[68] Li S, Li X, Li J, Deng X, Li Y (2007). Inhibition of oxidative-stress-induced platelet aggregation by androgen at physiological levels via its receptor is associated with the reduction of thromboxane A2 release from platelets. Steroids..

[69] Rodriguez G, Warkentin S, Risberg J, Rosadini G (1988). Sex differences in regional cerebral blood flow. J Cereb Blood Flow Metab.

[70] Ahnstedt H, Cao L, Krause DN, Warfvinge K, Saveland H, Nilsson OG (2013). Male-female differences in upregulation of vasoconstrictor responses in human cerebral arteries. PLoS One.

[71] De Silva TM, Broughton BR, Drummond GR, Sobey CG, Miller AA (2009). Gender influences cerebral vascular responses to angiotensin II through Nox2-derived reactive oxygen species. Stroke..

[72] Wang S, Zhang H, Liu Y, Li L, Guo Y, Jiao F, et al. Sex differences in the structure and function of rat middle cerebral arteries. Am J Phys Heart Circ Phys. 2020. 10.1152/ajpheart.00722.2019 PMC7346534 32216612

[73] WHO (2016). Global health estimates 2015: deaths by cause, age, sex, by country and by region, 2000-2015.

[74] Yang Q, Tong X, Schieb L, Vaughan A, Gillespie C, Wiltz JL (2017). Vital signs: recent trends in stroke death rates - United States, 2000-2015. MMWR Morb Mortal Wkly Rep.

[75] Bamford J, Dennis M, Sandercock P, Burn J, Warlow C (1990). The frequency, causes and timing of death within 30 days of a first stroke: the Oxfordshire Community Stroke Project. J Neurol Neurosurg Psychiatry.

[76] Appelros P, Stegmayr B, Terent A (2009). Sex differences in stroke epidemiology: a systematic review. Stroke..

[77] Liu F, McCullough LD (2012). Interactions between age, sex, and hormones in experimental ischemic stroke. Neurochem Int.

[78] Roy-O'Reilly M, McCullough LD (2018). Age and sex are critical factors in ischemic stroke pathology. Endocrinology..

[79] Fullerton HJ, Wu YW, Zhao S, Johnston SC (2003). Risk of stroke in children: ethnic and gender disparities. Neurology..

[80] Quillinan N, Deng G, Grewal H, Herson PS (2014). Androgens and stroke: good, bad or indifferent?. Exp Neurol.

[81] Normann S, de Veber G, Fobker M, Langer C, Kenet G, Bernard TJ (2009). Role of endogenous testosterone concentration in pediatric stroke. Ann Neurol.

[82] Vannucci SJ, Hurn PD (2009). Gender differences in pediatric stroke: is elevated testosterone a risk factor for boys?. Ann Neurol.

[83] Yeap BB, Hyde Z, Almeida OP, Norman PE, Chubb SA, Jamrozik K (2009). Lower testosterone levels predict incident stroke and transient ischemic attack in older men. J Clin Endocrinol Metab.

[84] Yeap BB, Alfonso H, Chubb SA, Hankey GJ, Handelsman DJ, Golledge J (2014). In older men, higher plasma testosterone or dihydrotestosterone is an independent predictor for reduced incidence of stroke but not myocardial infarction. J Clin Endocrinol Metab.

[85] Hollander M, Hak AE, Koudstaal PJ, Bots ML, Grobbee DE, Hofman A (2003). Comparison between measures of atherosclerosis and risk of stroke: the Rotterdam Study. Stroke..

[86] Jeppesen LL, Jorgensen HS, Nakayama H, Raaschou HO, Olsen TS, Winther K (1996). Decreased serum testosterone in men with acute ischemic stroke. Arterioscler Thromb Vasc Biol.

[87] Chen DY, See LC, Liu JR, Chuang CK, Pang ST, Hsieh IC (2017). Risk of cardiovascular ischemic events after surgical castration and gonadotropin-releasing hormone agonist therapy for prostate cancer: a nationwide cohort study. J Clin Oncol.

[88] Basaria S, Coviello AD, Travison TG, Storer TW, Farwell WR, Jette AM (2010). Adverse events associated with testosterone administration. N Engl J Med.

[89] Cortes YI, Barinas-Mitchell E, Suder Egnot N, Bhasin S, Jasuja R, Santoro N, et al. Associations of endogenous sex hormones with carotid plaque burden and characteristics in midlife women. J Clin Endocrinol Metab. 2020. 10.1210/clinem/dgz327 PMC7077951 31900485

[90] Gong Y, Xiao H, Li C, Bai J, Cheng X, Jin M (2013). Elevated t/e2 ratio is associated with an increased risk of cerebrovascular disease in elderly men. PLoS One.

[91] Zhao D, Guallar E, Ouyang P, Subramanya V, Vaidya D, Ndumele CE (2018). Endogenous sex hormones and incident cardiovascular disease in post-menopausal women. J Am Coll Cardiol.

[92] Alkayed NJ, Harukuni I, Kimes AS, London ED, Traystman RJ, Hurn PD (1998). Gender-linked brain injury in experimental stroke. Stroke..

[93] Faber JE, Moore SM, Lucitti JL, Aghajanian A, Zhang H (2017). Sex differences in the cerebral collateral circulation. Transl Stroke Res.

[94] Hall ED, Pazara KE, Linseman KL (1991). Sex differences in postischemic neuronal necrosis in gerbils. J Cereb Blood Flow Metab.

[95] Liu M, Dziennis S, Hurn PD, Alkayed NJ (2009). Mechanisms of gender-linked ischemic brain injury. Restor Neurol Neurosci.

[96] Manwani B, Liu F, Scranton V, Hammond MD, Sansing LH, McCullough LD (2013). Differential effects of aging and sex on stroke induced inflammation across the lifespan. Exp Neurol.

[97] McCullough LD, Zeng Z, Blizzard KK, Debchoudhury I, Hurn PD (2005). Ischemic nitric oxide and poly (ADP-ribose) polymerase-1 in cerebral ischemia: male toxicity, female protection. J Cereb Blood Flow Metab.

[98] Zhang W, Iliff JJ, Campbell CJ, Wang RK, Hurn PD, Alkayed NJ (2009). Role of soluble epoxide hydrolase in the sex-specific vascular response to cerebral ischemia. J Cereb Blood Flow Metab.

[99] De Vries GJ, Rissman EF, Simerly RB, Yang LY, Scordalakes EM, Auger CJ (2002). A model system for study of sex chromosome effects on sexually dimorphic neural and behavioral traits. J Neurosci.

[100] Manwani B, Bentivegna K, Benashski SE, Venna VR, Xu Y, Arnold AP (2015). Sex differences in ischemic stroke sensitivity are influenced by gonadal hormones, not by sex chromosome complement. J Cereb Blood Flow Metab.

[101] Uchida M, Palmateer JM, Herson PS, DeVries AC, Cheng J, Hurn PD (2009). Dose-dependent effects of androgens on outcome after focal cerebral ischemia in adult male mice. J Cereb Blood Flow Metab.

[102] Cheng J, Hu W, Toung TJ, Zhang Z, Parker SM, Roselli CE (2009). Age-dependent effects of testosterone in experimental stroke. J Cereb Blood Flow Metab.

[103] Hawk T, Zhang YQ, Rajakumar G, Day AL, Simpkins JW (1998). Testosterone increases and estradiol decreases middle cerebral artery occlusion lesion size in male rats. Brain Res.

[104] Yang SH, Perez E, Cutright J, Liu R, He Z, Day AL, et al. Testosterone increases neurotoxicity of glutamate in vitro and ischemia-reperfusion injury in an animal model. J Appl Physiol (1985). 2002;92(1):195-201. 10.1152/jappl.2002.92.1.195 11744660

[105] McCullough LD, Mirza MA, Xu Y, Bentivegna K, Steffens EB, Ritzel R (2016). Stroke sensitivity in the aged: sex chromosome complement vs. gonadal hormones. Aging (Albany NY).

[106] Pan Y, Zhang H, Acharya AB, Patrick PH, Oliver D, Morley JE (2005). Effect of testosterone on functional recovery in a castrate male rat stroke model. Brain Res.

[107] Leys D, Henon H, Mackowiak-Cordoliani MA, Pasquier F (2005). Poststroke dementia. Lancet Neurol.

[108] Goodman RA, Lochner KA, Thambisetty M, Wingo TS, Posner SF, Ling SM (2017). Prevalence of dementia subtypes in United States Medicare fee-for-service beneficiaries, 2011-2013. Alzheimers Dement.

[109] Attems J, Jellinger KA (2014). The overlap between vascular disease and Alzheimer’s disease--lessons from pathology. BMC Med.

[110] Toledo JB, Arnold SE, Raible K, Brettschneider J, Xie SX, Grossman M (2013). Contribution of cerebrovascular disease in autopsy confirmed neurodegenerative disease cases in the National Alzheimer’s Coordinating Centre. Brain..

[111] Copeland JR, McCracken CF, Dewey ME, Wilson KC, Doran M, Gilmore C (1999). Undifferentiated dementia, Alzheimer’s disease and vascular dementia: age- and gender-related incidence in Liverpool. The MRC-ALPHA Study Br J Psychiatry.

[112] Fujishima M, Kiyohara Y (2002). Incidence and risk factors of dementia in a defined elderly Japanese population: the Hisayama study. Ann N Y Acad Sci.

[113] Imfeld P, Bodmer M, Schuerch M, Jick SS, Meier CR (2013). Risk of incident stroke in patients with Alzheimer disease or vascular dementia. Neurology..

[114] Ruitenberg A, Ott A, van Swieten JC, Hofman A, Breteler MM (2001). Incidence of dementia: does gender make a difference?. Neurobiol Aging.

[115] Di Carlo A, Baldereschi M, Amaducci L, Lepore V, Bracco L, Maggi S (2002). Incidence of dementia, Alzheimer’s disease, and vascular dementia in Italy. The ILSA Study. J Am Geriatr Soc.

[116] Lobo A, Launer LJ, Fratiglioni L, Andersen K, Di Carlo A, Breteler MM (2000). Prevalence of dementia and major subtypes in Europe: a collaborative study of population-based cohorts. Neurologic Diseases in the Elderly Research Group. Neurology..

[117] Moffat SD, Zonderman AB, Metter EJ, Blackman MR, Harman SM, Resnick SM (2002). Longitudinal assessment of serum free testosterone concentration predicts memory performance and cognitive status in elderly men. J Clin Endocrinol Metab.

[118] Ravaglia G, Forti P, Maioli F, Chiappelli M, Montesi F, Tumini E (2007). Blood inflammatory markers and risk of dementia: the conselice study of brain aging. Neurobiol Aging.

[119] Geerlings MI, Strozyk D, Masaki K, Remaley AT, Petrovitch H, Ross GW (2006). Endogenous sex hormones, cognitive decline, and future dementia in old men. Ann Neurol.

[120] Ford AH, Yeap BB, Flicker L, Hankey GJ, Chubb SAP, Golledge J (2018). Sex hormones and incident dementia in older men: the health in men study. Psychoneuroendocrinology..

[121] Tsai HH, Niu J, Munji R, Davalos D, Chang J, Zhang H (2016). Oligodendrocyte precursors migrate along vasculature in the developing nervous system. Science..

[122] Smith C, Contreras-Garza J, Cunningham RL, Wong JM, Vann PH, Metzger D, et al. Chronic testosterone deprivation sensitizes the middle-aged rat brain to damaging effects of testosterone replacement. Neuroendocrinology. 2019. 10.1159/000504445 PMC12786679 31671430

[123] Ahlbom E, Grandison L, Bonfoco E, Zhivotovsky B, Ceccatelli S (1999). Androgen treatment of neonatal rats decreases susceptibility of cerebellar granule neurons to oxidative stress in vitro. Eur J Neurosci.

[124] Ahlbom E, Prins GS, Ceccatelli S (2001). Testosterone protects cerebellar granule cells from oxidative stress-induced cell death through a receptor mediated mechanism. Brain Res.

[125] Pike CJ (2001). Testosterone attenuates beta-amyloid toxicity in cultured hippocampal neurons. Brain Res.

[126] Hammond J, Le Q, Goodyer C, Gelfand M, Trifiro M, LeBlanc A (2001). Testosterone-mediated neuroprotection through the androgen receptor in human primary neurons. J Neurochem.

[127] Schmidt AJ, Krieg J, Vedder H (2002). Differential effects of glucocorticoids and gonadal steroids on glutathione levels in neuronal and glial cell systems. J Neurosci Res.

[128] Fanaei H, Karimian SM, Sadeghipour HR, Hassanzade G, Kasaeian A, Attari F (2014). Testosterone enhances functional recovery after stroke through promotion of antioxidant defenses, BDNF levels and neurogenesis in male rats. Brain Res.

[129] Snapyan M, Lemasson M, Brill MS, Blais M, Massouh M, Ninkovic J (2009). Vasculature guides migrating neuronal precursors in the adult mammalian forebrain via brain-derived neurotrophic factor signaling. J Neurosci Off J Soc Neurosci.

[130] Garcia-Diaz B, Bachelin C, Coulpier F, Gerschenfeld G, Deboux C, Zujovic V (2019). Blood vessels guide Schwann cell migration in the adult demyelinated CNS through Eph/ephrin signaling. Acta Neuropathol.

[131] Abi Ghanem C, Degerny C, Hussain R, Liere P, Pianos A, Tourpin S (2017). Long-lasting masculinizing effects of postnatal androgens on myelin governed by the brain androgen receptor. PLoS Genet.

[132] Bielecki B, Mattern C, Ghoumari AM, Javaid S, Smietanka K, Abi Ghanem C (2016). Unexpected central role of the androgen receptor in the spontaneous regeneration of myelin. Proc Natl Acad Sci U S A.

[133] Sheng XG, Feng JZ, Wu S, Jin LJ, Yu XY, Zhang B (2004). Differentiation of rabbit bone marrow mesenchymal stem cells into myogenic cells in vitro and expression of vascular endothelial growth factor gene after transfection. Di Yi Jun Yi Da Xue Xue Bao.

[134] Sheng G, Xu X, Lin YF, Wang CE, Rong J, Cheng D (2008). Huntingtin-associated protein 1 interacts with Ahi1 to regulate cerebellar and brainstem development in mice. J Clin Invest.

[135] Kokovay E, Goderie S, Wang Y, Lotz S, Lin G, Sun Y (2010). Adult SVZ lineage cells home to and leave the vascular niche via differential responses to SDF1/CXCR4 signaling. Cell Stem Cell.

[136] Spritzer MD, Galea LA (2007). Testosterone and dihydrotestosterone, but not estradiol, enhance survival of new hippocampal neurons in adult male rats. Dev Neurobiol.

[137] Hamson DK, Wainwright SR, Taylor JR, Jones BA, Watson NV, Galea LA (2013). Androgens increase survival of adult-born neurons in the dentate gyrus by an androgen receptor-dependent mechanism in male rats. Endocrinology..

[138] Swift-Gallant A, Duarte-Guterman P, Hamson DK, Ibrahim M, Monks DA, Galea LAM (2018). Neural androgen receptors affect the number of surviving new neurones in the adult dentate gyrus of male mice. J Neuroendocrinol.

[139] Gannon OJ, Robison LS, Custozzo AJ, Zuloaga KL (2019). Sex differences in risk factors for vascular contributions to cognitive impairment & dementia. Neurochem Int.

[140] Gems D (2014). Evolution of sexually dimorphic longevity in humans. Aging (Albany NY).

[141] Feldman HA, Longcope C, Derby CA, Johannes CB, Araujo AB, Coviello AD (2002). Age trends in the level of serum testosterone and other hormones in middle-aged men: longitudinal results from the Massachusetts male aging study. J Clin Endocrinol Metab.

[142] Harman SM, Metter EJ, Tobin JD, Pearson J, Blackman MR (2001). Baltimore Longitudinal Study of A. Longitudinal effects of aging on serum total and free testosterone levels in healthy men. Baltimore Longitudinal Study of Aging. J Clin Endocrinol Metab.

[143] Goodman-Gruen D, Barrett-Connor E (2000). Sex differences in the association of endogenous sex hormone levels and glucose tolerance status in older men and women. Diabetes Care.

[144] Kostakis EK, Gkioni LN, Macut D, Mastorakos G (2019). Androgens in menopausal women: not only polycystic ovary syndrome. Front Horm Res.

[145] Zumoff B, Strain GW, Miller LK, Rosner W (1995). Twenty-four-hour mean plasma testosterone concentration declines with age in normal premenopausal women. J Clin Endocrinol Metab.

[146] Laughlin GA, Barrett-Connor E, Kritz-Silverstein D, von Muhlen D (2000). Hysterectomy, oophorectomy, and endogenous sex hormone levels in older women: the Rancho Bernardo Study. J Clin Endocrinol Metab.

[147] Moreau KL, Babcock MC, Hildreth KL (2020). Sex differences in vascular aging in response to testosterone. Biol Sex Differ.

[148] Diamanti-Kandarakis E (2008). Polycystic ovarian syndrome: pathophysiology, molecular aspects and clinical implications. Expert Rev Mol Med.

[149] De Lima LG, Soares BG, Saconato H, Atallah AN, da Silva EM (2013). Beta-blockers for preventing stroke recurrence. Cochrane Database Syst Rev.

[150] Rees DA, Udiawar M, Berlot R, Jones DK, O’Sullivan MJ (2016). White matter microstructure and cognitive function in young women with polycystic ovary syndrome. J Clin Endocrinol Metab.

[151] Yamada M, Sasaki H, Mimori Y, Kasagi F, Sudoh S, Ikeda J (1999). Prevalence and risks of dementia in the Japanese population: RERF’s adult health study Hiroshima subjects. Radiation Effects Research Foundation. J Am Geriatr Soc.

[152] Gillis EE, Sullivan JC (2016). Sex differences in hypertension: recent advances. Hypertension..

[153] Khaw KT, Barrett-Connor E (1988). Blood pressure and endogenous testosterone in men: an inverse relationship. J Hypertens.

[154] Ziemens B, Wallaschofski H, Volzke H, Rettig R, Dorr M, Nauck M (2013). Positive association between testosterone, blood pressure, and hypertension in women: longitudinal findings from the Study of Health in Pomerania. J Hypertens.

[155] Ling S, Komesaroff PA, Sudhir K (2009). Cardiovascular physiology of androgens and androgen testosterone therapy in postmenopausal women. Endocr Metab Immune Disord Drug Targets.

[156] Carroll MD, Fryar CD, Nguyen DT (2017). Total and high-density lipoprotein cholesterol in adults: United States, 2015-2016. NCHS Data Brief.

[157] Ancelin ML, Ripoche E, Dupuy AM, Barberger-Gateau P, Auriacombe S, Rouaud O (2013). Sex differences in the associations between lipid levels and incident dementia. Journal of Alzheimer's disease : JAD.

[158] Traish AM, Abdou R, Kypreos KE (2009). Androgen deficiency and atherosclerosis: the lipid link. Vasc Pharmacol.

[159] Monroe AK, Dobs AS (2013). The effect of androgens on lipids. Curr Opin Endocrinol Diabetes Obes.

[160] Goh HH, Loke DF, Ratnam SS (1995). The impact of long-term testosterone replacement therapy on lipid and lipoprotein profiles in women. Maturitas..

[161] Chandra AK, Ghosh R, Chatterjee A, Sarkar M (2010). Protection against vanadium-induced testicular toxicity by testosterone propionate in rats. Toxicol Mech Methods.

[162] Irwig MS (2018). Cardiovascular health in transgender people. Rev Endocr Metab Disord.

[163] Livingstone SJ, Levin D, Looker HC, Lindsay RS, Wild SH, Joss N (2015). Estimated life expectancy in a Scottish cohort with type 1 diabetes, 2008-2010. Jama..

[164] Soedamah-Muthu SS, Fuller JH, Mulnier HE, Raleigh VS, Lawrenson RA, Colhoun HM (2006). All-cause mortality rates in patients with type 1 diabetes mellitus compared with a non-diabetic population from the UK general practice research database, 1992-1999. Diabetologia..

[165] Secrest AM, Prince CT, Costacou T, Miller RG, Orchard TJ (2013). Predictors of and survival after incident stroke in type 1 diabetes. Diab Vasc Dis Res.

[166] Saczynski JS, Jonsdottir MK, Garcia ME, Jonsson PV, Peila R, Eiriksdottir G (2008). Cognitive impairment: an increasingly important complication of type 2 diabetes: the age, gene/environment susceptibility--Reykjavik study. Am J Epidemiol.

[167] Ahtiluoto S, Polvikoski T, Peltonen M, Solomon A, Tuomilehto J, Winblad B (2010). Diabetes, Alzheimer disease, and vascular dementia: a population-based neuropathologic study. Neurology..

[168] Peila R, Rodriguez BL, Launer LJ, Honolulu-Asia Aging S (2002). Type 2 diabetes, APOE gene, and the risk for dementia and related pathologies: the Honolulu-Asia Aging Study. Diabetes..

[169] Chatterjee S, Peters SA, Woodward M, Mejia Arango S, Batty GD, Beckett N (2016). Type 2 diabetes as a risk factor for dementia in women compared with men: a pooled analysis of 2.3 million people comprising more than 100,000 cases of dementia. Diabetes Care.

[170] Liu CL, Lin MY, Hwang SJ, Liu CK, Lee HL, Wu MT (2018). Factors associated with type 2 diabetes in patients with vascular dementia: a population-based cross-sectional study. BMC Endocr Disord.

[171] Kautzky-Willer A, Harreiter J, Pacini G (2016). Sex and gender differences in risk, pathophysiology and complications of type 2 diabetes mellitus. Endocr Rev.

[172] Yao QM, Wang B, An XF, Zhang JA, Ding L (2018). Testosterone level and risk of type 2 diabetes in men: a systematic review and meta-analysis. Endocr Connect.

[173] Rovira-Llopis S, Banuls C, de Maranon AM, Diaz-Morales N, Jover A, Garzon S (2017). Low testosterone levels are related to oxidative stress, mitochondrial dysfunction and altered subclinical atherosclerotic markers in type 2 diabetic male patients. Free Radic Biol Med.

[174] Navarro G, Allard C, Xu W, Mauvais-Jarvis F (2015). The role of androgens in metabolism, obesity, and diabetes in males and females. Obesity (Silver Spring).

[175] Sabayan B, Foroughinia F, Haghighi AB, Mowla A (2007). Are women with polycystic ovary syndrome (PCOS) at higher risk for development of alzheimer disease?. Alzheimer Dis Assoc Disord.

[176] Ohara T, Doi Y, Ninomiya T, Hirakawa Y, Hata J, Iwaki T (2011). Glucose tolerance status and risk of dementia in the community: the Hisayama study. Neurology..

[177] Bodenant M, Kuulasmaa K, Wagner A, Kee F, Palmieri L, Ferrario MM (2011). Measures of abdominal adiposity and the risk of stroke: the MOnica Risk, Genetics, Archiving and Monograph (MORGAM) study. Stroke..

[178] Kim J, Yoon SJ, Woo MH, Kim SH, Kim NK, Kim J (2017). Differential impact of serum total bilirubin level on cerebral atherosclerosis and cerebral small vessel disease. PLoS One.

[179] Whitmer RA, Gunderson EP, Quesenberry CP, Zhou J, Yaffe K (2007). Body mass index in midlife and risk of Alzheimer disease and vascular dementia. Curr Alzheimer Res.

[180] Ahlskog JE, Geda YE, Graff-Radford NR, Petersen RC (2011). Physical exercise as a preventive or disease-modifying treatment of dementia and brain aging. Mayo Clin Proc.

[181] Morris MC, Tangney CC (2014). Dietary fat composition and dementia risk. Neurobiol Aging.

[182] Hales CM, Fryar CD, Carroll MD, Freedman DS, Ogden CL (2018). Trends in obesity and severe obesity prevalence in US youth and adults by sex and age, 2007-2008 to 2015-2016. Jama..

[183] Lukanova A, Lundin E, Zeleniuch-Jacquotte A, Muti P, Mure A, Rinaldi S (2004). Body mass index, circulating levels of sex-steroid hormones, IGF-I and IGF-binding protein-3: a cross-sectional study in healthy women. Eur J Endocrinol.

[184] Pasquali R, Oriolo C (2019). Obesity and androgens in women. Front Horm Res.

[185] Salinero AE, Anderson BM, Zuloaga KL (2018). Sex differences in the metabolic effects of diet-induced obesity vary by age of onset. Int J Obes.

[186] Kelly DM, Jones TH (2015). Testosterone and obesity. Obes Rev.

[187] Malkin CJ, Pugh PJ, Morris PD, Kerry KE, Jones RD, Jones TH (2004). Testosterone replacement in hypogonadal men with angina improves ischaemic threshold and quality of life. Heart..

[188] Stanworth R, Jones T (2009). Testosterone in obesity, metabolic syndrome and type 2 diabetes. Front Horm Res.

[189] Pitteloud N, Dwyer AA, DeCruz S, Lee H, Boepple PA, Crowley WF (2008). The relative role of gonadal sex steroids and gonadotropin-releasing hormone pulse frequency in the regulation of follicle-stimulating hormone secretion in men. J Clin Endocrinol Metab.

[190] Hamilton EJ, Gianatti E, Strauss BJ, Wentworth J, Lim-Joon D, Bolton D (2011). Increase in visceral and subcutaneous abdominal fat in men with prostate cancer treated with androgen deprivation therapy. Clin Endocrinol.

[191] Grossmann M, Ng Tang Fui M, Cheung AS. Late-onset hypogonadism: metabolic impact. Andrology. 2019. 10.1111/andr.12705 31502758

[192] Fernandez CJ, Chacko EC, Pappachan JM (2019). Male obesity-related secondary hypogonadism - pathophysiology, clinical implications and management. Eur Endocrinol.

[193] Harada N (2018). Role of androgens in energy metabolism affecting on body composition, metabolic syndrome, type 2 diabetes, cardiovascular disease, and longevity: lessons from a meta-analysis and rodent studies. Biosci Biotechnol Biochem.

[194] Mountain DJ, Freeman MB, Kirkpatrick SS, Cook RB, Chalk JE, Stevens SL (2013). Effect of hormone replacement therapy in matrix metalloproteinase expression and intimal hyperplasia development after vascular injury. Ann Vasc Surg.

